# Beating the break-even point with a discrete-variable-encoded logical qubit

**DOI:** 10.1038/s41586-023-05784-4

**Published:** 2023-03-22

**Authors:** Zhongchu Ni, Sai Li, Xiaowei Deng, Yanyan Cai, Libo Zhang, Weiting Wang, Zhen-Biao Yang, Haifeng Yu, Fei Yan, Song Liu, Chang-Ling Zou, Luyan Sun, Shi-Biao Zheng, Yuan Xu, Dapeng Yu

**Affiliations:** 1 Shenzhen Institute for Quantum Science and Engineering, Southern University of Science and Technology, Shenzhen, China; 2 Guangdong Provincial Key Laboratory of Quantum Science and Engineering, Southern University of Science and Technology, Shenzhen, China; 3 Department of Physics, Southern University of Science and Technology, Shenzhen, China; 4 Center for Quantum Information, Institute for Interdisciplinary Information Sciences, Tsinghua University, Beijing, China; 5 Fujian Key Laboratory of Quantum Information and Quantum Optics, College of Physics and Information Engineering, Fuzhou University, Fuzhou, China; 6 Beijing Academy of Quantum Information Sciences, Beijing, China; 7 International Quantum Academy, and Shenzhen Branch, Hefei National Laboratory, Shenzhen, China; 8 CAS Key Laboratory of Quantum Information, University of Science and Technology of China, Hefei, China; 9 Hefei National Laboratory, Hefei, China

**Keywords:** Quantum information, Qubits, Quantum optics

## Abstract

Quantum error correction (QEC) aims to protect logical qubits from noises by using the redundancy of a large Hilbert space, which allows errors to be detected and corrected in real time^1^. In most QEC codes^2–8^, a logical qubit is encoded in some discrete variables, for example photon numbers, so that the encoded quantum information can be unambiguously extracted after processing. Over the past decade, repetitive QEC has been demonstrated with various discrete-variable-encoded scenarios^9–17^. However, extending the lifetimes of thus-encoded logical qubits beyond the best available physical qubit still remains elusive, which represents a break-even point for judging the practical usefulness of QEC. Here we demonstrate a QEC procedure in a circuit quantum electrodynamics architecture^18^, where the logical qubit is binomially encoded in photon-number states of a microwave cavity^8^, dispersively coupled to an auxiliary superconducting qubit. By applying a pulse featuring a tailored frequency comb to the auxiliary qubit, we can repetitively extract the error syndrome with high fidelity and perform error correction with feedback control accordingly, thereby exceeding the break-even point by about 16% lifetime enhancement. Our work illustrates the potential of hardware-efficient discrete-variable encodings for fault-tolerant quantum computation^19^.

## Abstract (web summary)

The lifetime of a qubit in a circuit quantum electrodynamics architecture can exceed the break-even point through quantum error correction, representing a key step towards scalable quantum computing.

## Main

One of the main obstacles for building a quantum computer is environmentally induced decoherence, which destroys the quantum information stored in the qubits. The errors caused by decoherence can be corrected by repetitive application of a quantum error correction (QEC) procedure, whereby the logical qubit is encoded in a high-dimensional Hilbert space, such that different errors project the system into different orthogonal subspaces and thus can be unambiguously identified and corrected without disturbing the stored quantum information. In conventional QEC schemes^1,9^, the code words of a logical qubit are formed by two highly symmetric entangled states of several physical qubits encoded with some discrete variables. The past two decades have witnessed remarkable advances in experimental demonstrations of this kind of QEC code in different systems, including nuclear spins^5,6^, nitrogen-vacancy centres in diamond^10,20^, trapped ions^7,11,21–23^, photonic qubits^24^, silicon spin qubits^25^ and superconducting circuits^12–16,26,27^. However, in these experiments, the lifetime of the logical qubit still needs to be greatly extended to reach that of the best available physical component, which is regarded as the break-even point for judging whether or not a QEC code can benefit quantum information storage and processing.

An alternative QEC encoding scheme is to use the large space of an oscillator, which can be used to encode either a continuous-variable or a discrete-variable qubit^28–32^. Both types of code can tolerate errors due to loss and gain of energy quanta, enabling QEC to be performed in a hardware-efficient way. Circuit quantum electrodynamics (QED) systems^18^ represent an ideal platform for realizing such encoding schemes: the break-even point has been exceeded in two breakthrough experiments^33,34^ by distributing the quantum information over an infinite-dimensional Hilbert space of a continuous-variable-encoded photonic qubit, but the code words of this photonic qubit are not strictly orthogonal. This inherent restriction can be overcome with discrete-variable encoding schemes, whereby the code words of a logical qubit are encoded with mutually orthogonal Fock states of an oscillator. This feature, together with their intrinsic compatibility with error-correctable gates^35,36^ and their usefulness in logically connecting modules in a quantum network^37^, makes such discrete-variable qubits promising in fault-tolerant quantum computation. These advantages can be turned into practical benefits in real quantum information processing only when the lifetime of the encoded logical qubits is extended beyond the break-even point, which, however, remains an elusive result, although enduring efforts have been made towards this goal^17,32^.

Here, we demonstrate the exceeding of the QEC break-even point by real-time feedback correction for a discrete-variable photonic qubit in a microwave cavity, whose code words remain mutually orthogonal and can be unambiguously discriminated. The dominant error, single photon loss, of the logical qubit is mapped to the state of a Josephson junction-based nonlinear oscillator that is dispersively coupled to the cavity and serves as an auxiliary qubit, realized with a continuous pulse involving an ingeniously tailored comb of frequency components. As the driving frequencies aim at the error space where a photon loss event occurs, perturbations on the logical qubit are highly suppressed when it remains in the encoded logical space. Another intrinsic advantage of this error syndrome detection is that the continuous driving protects the system from the auxiliary qubit’s dephasing noise. We demonstrate this procedure with the lowest-order binomial code and extend the stored quantum information lifetime 16% longer than the best physical qubit, encoded in the two lowest Fock states and referred to as the Fock qubit. A more important characteristic associated with this error-detecting procedure is that neither the logical nor the error space needs to have a definite parity, which allows the implementation of QEC codes that can tolerate losses of more than one photon.

**Fig. 1:**
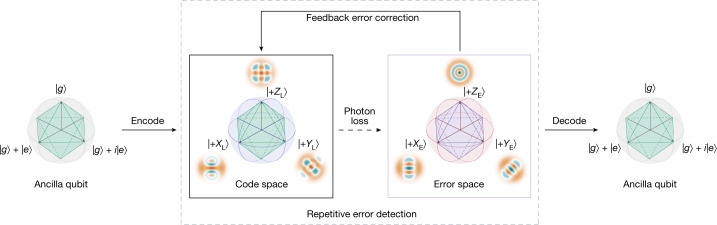
Schematic of the QEC procedure with the lowest-order binomially encoded logical qubit. The auxiliary qubit is first encoded to the logical qubit in an oscillator with $\{\left|{0}_{{\rm{L}}}\right\rangle =\left(\left|0\right\rangle +\left|4\right\rangle \right)/\sqrt{2},\left|{1}_{{\rm{L}}}\right\rangle =\left|2\right\rangle \}$. Once a single-photon-jump error occurs, the logical qubit state falls out of the code space to the error space with the basis states: $\{\left|{0}_{{\rm{E}}}\right\rangle =\left|3\right\rangle ,\left|{1}_{{\rm{E}}}\right\rangle =\left|1\right\rangle \}$. After repetitive error detecting and correcting, the logical qubit state is protected against single-photon-jump errors. Finally, quantum state is decoded back to the auxiliary qubit for a final state characterization. The cardinal point states in the Bloch spheres of the code and error spaces are defined as $\left|+{Z}_{{\rm{L}}({\rm{E}})}\right\rangle =\left|{0}_{{\rm{L}}({\rm{E}})}\right\rangle ,\left|+{X}_{{\rm{L}}({\rm{E}})}\right\rangle =(\left|{0}_{{\rm{L}}({\rm{E}})}\right\rangle +\left|{1}_{{\rm{L}}({\rm{E}})}\right\rangle )/\sqrt{2}$ and $\left|+{Y}_{{\rm{L}}({\rm{E}})}\right\rangle =(\left|{0}_{{\rm{L}}({\rm{E}})}\right\rangle +i\left|{1}_{{\rm{L}}({\rm{E}})}\right\rangle )/\sqrt{2}$, respectively.

The key stages of a QEC procedure are encoding the quantum information to the logical qubit from the auxiliary qubit, the error syndrome measurement, the real-time error correction of the system depending on the measurement output and the decoding process to read out the quantum information stored in the logical qubit. Our logical qubit is realized in a three-dimensional microwave cavity, and the dominant decoherence to combat is the excitation loss error. The logical qubit is encoded with a binomial code^8^, with the code words:

$$\begin{array}{r}| {0}_{{\rm{L}}}\rangle =(| 0\rangle +| 4\rangle )/\sqrt{2},\\ | {1}_{{\rm{L}}}\rangle =| 2\rangle ,\end{array}$$

where the number in each ket denotes the photon number in the cavity. The binomial code is a typical stabilizer QEC code: when the single-photon-loss error occurs, the quantum information is projected into the error space spanned by $\{\left|{0}_{{\rm{E}}}\right\rangle =\left|3\right\rangle ,\left|{1}_{{\rm{E}}}\right\rangle =\left|1\right\rangle \}$, with the photon number parity acting as the error syndrome to distinguish these two spaces. A general QEC protection of quantum information stored in the bosonic system is illustrated in Fig. 1. After correctly measuring the photon number parity and applying the corresponding correction operations in real time, quantum information stored in the cavity can be recovered.

The experiments are performed with a circuit QED architecture^18^, where a superconducting transmon qubit^38^ as an auxiliary qubit is dispersively coupled to a three-dimensional microwave cavity^39–41^. The auxiliary qubit has an energy relaxation time of about 98 μs and a pure dephasing time of 968 μs, whereas the storage cavity has a single-photon lifetime of 578 μs (corresponding to a decay rate *κ*_s_/2π = 0.28 kHz) and a pure dephasing time of 4.4 ms. The universal control of the multiple photon states of the cavity can be realized by using the anharmonicity of the auxiliary qubit, and thus the key stages of the QEC procedure, as illustrated in Fig. 1, can be achieved by encoding the logical qubit in the high-dimensional Fock spaces of the bosonic mode.

**Fig. 2:**
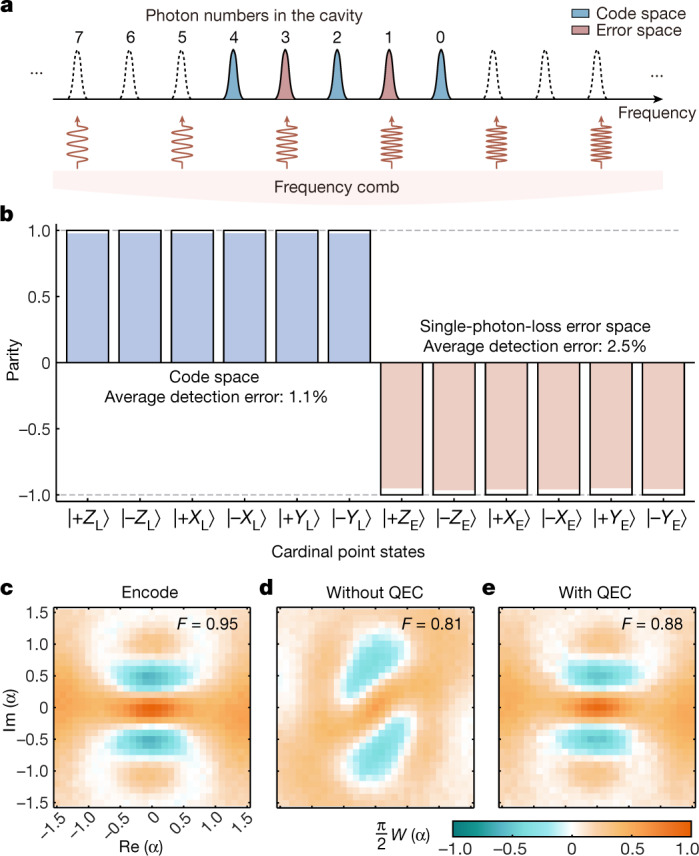
Frequency comb control to measure the error syndrome. **a**, Frequency comb control is realized by mapping the photon number parity of the logical state to the auxiliary qubit state by applying a microwave pulse with multi-frequency components to the auxiliary qubit. Two components match the auxiliary qubit frequencies when the logical qubit is in the error space and other components are chosen symmetrically for the code space to eliminate the off-resonant driving effect on the logical states. **b**, Bar chart of the measured photon number parities for the six cardinal point states on the Bloch spheres of the logical qubit in the code and error spaces with the frequency comb parity measurement. Solid black frames correspond to the ideal parities ± 1 for the logical states in the code and error spaces. The numbers represent the average parity detection errors in these two spaces. **c**, Measured Wigner function of the cavity state after encoding the logical qubit in the $\left|+{X}_{{\rm{L}}}\right\rangle$ state. **d**,**e**, Measured Wigner functions of the same cavity state after a waiting time of about 90 μs without (**d**) and with (**e**) a single QEC operation. The numbers in these Wigner functions represent the corresponding state fidelities. Source Data

Our route towards the break-even points in the QEC is twofold: improving both the operation fidelity to the logical qubit and the error syndrome measurement fidelity. The first goal is achieved by using a tantalum transmon qubit with high coherence^42,43^ and an optimal quantum control technique^44^ with carefully calibrated system parameters (Methods). We attempt the second goal by an ingenious scheme of projection measurement of a selected collection of Fock states. The principle of the scheme is illustrated in Fig. 2a, where a classical microwave pulse containing 2*M* frequency components is applied on the auxiliary qubit to read out the Fock states. Because the frequency of the auxiliary qubit depends on the photon number *n* (see Methods for more details), error syndrome detection is achieved by mapping the even parity to the auxiliary qubit ground state $\left|g\right\rangle$ (and the odd parity to the excited state $\left|e\right\rangle$) in a quantum non-demolition manner. This approach holds potential advantages of more flexible choices of error spaces and less sensitivity to auxiliary qubit damping and dephasing errors because the auxiliary qubit excitation is pronounced only when loss error occurs.

To characterize our syndrome measurement, the cavity is encoded to the six cardinal point states in the Bloch spheres of both the code and error spaces on the basis of the lowest-order binomial code words. The measured results of the cavity photon number parities are presented in Fig. 2b and show an average detection error of 1.1% and 2.5% for the cavity states in the code and error spaces, respectively. The encoding of the cavity, one of the most elementary processes of QEC, is further verified by the Wigner function with a high fidelity of 0.95, as shown in Fig. 2c.

On the basis of the above techniques, the QEC process of the binomial code can be implemented following the procedure in Fig. 1. However, practical imperfections limit the QEC performance: (1) during a waiting time of *t*_w_, that is, an idle process, there is a probability of about $2{({\kappa }_{{\rm{s}}}{t}_{{\rm{w}}})}^{2}\exp (-2{\kappa }_{{\rm{s}}}{t}_{{\rm{w}}})$ of a two-photon-loss error, which is undetectable for this lowest-order binomial code. (2) Owing to the non-commutativity of the single-photon-loss error and the self-Kerr interaction of the cavity, there is a large dephasing effect of the logical qubit induced by the unpredictable photon loss event, thus destroying the stored quantum information. (3) Quantum recovery operations are imperfect. It is worth noting that there is a logical state distortion even if no photon loss is detected^8^. Strategies to mitigate the above imperfections are introduced, taking into account the whole system: choose an optimal waiting time, use a two-layer QEC procedure^17^ to avoid unnecessary operation errors introduced by the error corrections and adopt the photon-number-resolved a.c. Stark shift (PASS) method^35^ during idle operations to suppress photon-jump-error-induced decoherence in the code space (see Supplementary Information for more details). The measured Wigner functions of the cavity states after a single QEC cycle (about 90 μs of waiting) without and with performing the error correction operation are shown in Fig. 2d,e, with state fidelities of 0.81 and 0.88, respectively.

**Fig. 3:**
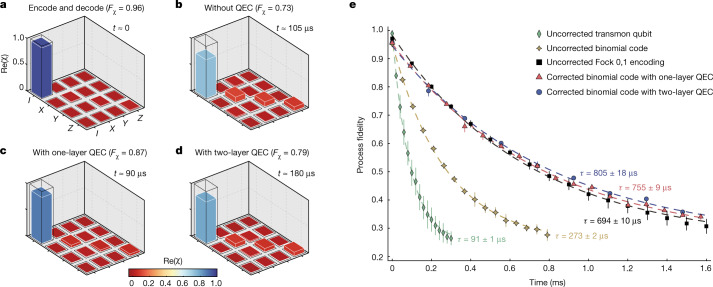
Performance of repetitive QEC operations. **a**–**d**, Bar charts of the real parts of the process matrices for an encode and decode process (**a**), a waiting time of about 105 μs without QEC (**b**), a cycle time of about 90 μs with one-layer QEC operation (**c**) and a cycle time of about 180 μs with two-layer QEC operation (**d**). The numbers in brackets represent the process fidelities for each case. **e**, Process fidelity decays as a function of time for different encodings. Error bars correspond to 1 s.d. of several repeated measurements. The process fidelities for both the corrected binomial code with one-layer QEC (red triangles) and two-layer QEC (blue circles) exhibit slow decay, compared with the uncorrected Fock states $\{\left|0\right\rangle ,\left|1\right\rangle \}$ encoding (black squares), which defines the break-even point in this system. The corrected binomial code with two-layer QEC offers an improvement over the break-even point by a factor of 1.2, and also surpasses the uncorrected binomial code (yellow stars) by a factor of 2.9 and the uncorrected transmon qubit (green diamonds) by a factor of 8.8. All curves are fitted using *F*_*χ*_ = *Ae*^−*t*/*τ*^ + 0.25 to extract the lifetimes *τ* of the corresponding encodings. Uncertainties on *τ* are obtained from the fittings. Source Data

The performance of the QEC is benchmarked by the process fidelity ${F}_{\chi }$, which is defined as the trace of *χ*_exp_*χ*_ideal_, where *χ*_exp_ denotes the experimentally measured process matrix for the QEC process and *χ*_ideal_ is the ideal process matrix for an identity operation. In Fig. 3a, we present the measured process matrix for the encoding and decoding process only, which indicates a reference fidelity of 0.96. In the absence of a QEC operation after a waiting time of 105 μs, the process fidelity is reduced to a value of 0.73 because of the inability to protect the quantum information stored in the cavity from the single-photon-loss error, with the corresponding measured process matrix shown in Fig. 3b. When using the QEC operation, the process fidelity is improved because of the protection from the single-photon-loss error, with the process matrices for the one-layer and two-layer QECs shown in Fig. 3c,d, respectively.

The most important benchmark to characterize the performance of a QEC procedure is the gain in the lifetime of the protected logical qubit against that of the constituent element with the longest lifetime. For the three-dimensional circuit QED device, the best physical qubit is encoded with the two lowest photon-number states $\{\left|0\right\rangle ,\left|1\right\rangle \}$, which is more robust against decoherence effects than any other encoded photonic qubit without QEC protection. To quantitatively show the advantage of our QEC scheme, in Fig. 3e we present the measured process fidelities of the corrected binomial code as a function of the storage time with the repetitive one-layer (red triangles) and two-layer (blue circles) QECs, as well as those for the unprotected binomial code (yellow stars), the transmon qubit (green diamonds) and the Fock qubit (black squares) for comparison.

All curves are fitted according to the function *F*_*χ*_ = *Ae*^−*t*/*τ*^ + 0.25, with *τ* corresponding to the lifetime of the specific encoding and *A* being a fitting parameter. The offset in the fitting function is fixed to 0.25, implying a complete loss of information at the final time. As a result, the lifetime *τ* for the corrected binomial code with one-layer QEC is improved by about 8.3 times compared with the uncorrected transmon qubit and 2.8 times compared with the uncorrected binomial code. In particular, *τ* is improved to about 1.1 times that of the uncorrected Fock qubit encoding, that is, exceeding the break-even point of QEC in this system. Using the two-layer QEC scheme, the corresponding lifetime *τ* of the logical qubit is improved to about 8.8 times that of the uncorrected transmon qubit, 2.9 times that of the uncorrected binomial code and 1.2 times that of the break-even point. These results demonstrate that the quantum information stored in the cavity with multiphoton binomial encoding can indeed be preserved and protected from photon loss errors by means of repetitive QEC operations.

Table 1 shows an overall error analysis for the one-layer and two-layer QEC experiments. The error sources are divided into four parts: the intrinsic errors for the lowest-order binomial code, the error detection errors, the recovery operation errors and the auxiliary qubit thermal excitation errors during the QEC cycle. These errors can be estimated from either the numerical simulations or the measurement results of individual calibration experiments (Supplementary Information). The predicted lifetimes *τ* for the QEC experiments, calculated by $\tau =-{T}_{{\rm{w}}}/ln(1-{\epsilon })$^17^, with *T*_w_ and *ϵ* being the total duration and the weighted total error per QEC cycle, are consistent with those in our QEC experiments.

**Table 1:** Error budget for the one-layer and two-layer QEC processes. The predicted lifetime calculated from the error model agrees well with the measured lifetimes

Error source	One-layer QEC	Two-layer QEC
Intrinsic error^a^	6.4%	12.4%
Detection error	1.4%	2.8%
Recovery error	2.9%	3.8%
Thermal error	0.8%	1.1%
Total error	11.5%	20.1%
Predicted lifetime	757 μs	824 μs
Measured lifetime	755 ± 9 μs	805 ± 18 μs

^a^These errors are estimated from numerical simulations.

In conclusion, we experimentally demonstrate the prolonged coherence time of quantum information encoded with discrete variables in a bosonic mode by repetitive QEC. The break-even point has been reached by carefully designing the QEC procedure to balance the fidelity losses due to undetectable errors during the idle process, and error detection and correction operations. At present, the main infidelity is contributed by the two-photon-loss error that is beyond the ability of our current QEC code, but can be corrected by higher-order binomial codes^8^. Our frequency comb method could be used to measure the generalized photon number parity of such codes, enabling detection and correction of both single-photon-loss and two-photon-loss errors. Our work thus represents a key step towards scalable quantum computing and provides a practical guide for system optimization of quantum control and the design of the QEC procedure for future applications of logical qubits.

## Methods

### Experimental device and set-up

The circuit QED device in our experiment uses a hybrid three-dimensional–planar architecture^40^ and consists of a superconducting transmon qubit^38^, a coaxial stub cavity and a Purcell-filtered stripline readout resonator (see Fig. S1 in the Supplementary Information). The high-Q cavity is designed with a cylindrical re-entrant quarter-wave transmission line resonator^41^, and machined from high-purity (99.9995%) aluminium. A horizontal tunnel is used to house a sapphire chip, on which the antenna pads of the transmon qubit and the striplines of the low-Q readout resonator are patterned with a thin tantalum film^42,43^. The single Al-AlO_x_-Al trilayer Josephson junction of the transmon qubit is fabricated using a double-angle evaporation technique.

The fast feedback control is implemented with Zurich Instruments UHFQA and HDAWG, which are connected to each other through a digital input/output (DIO) link cable for real-time feedback control. The UHFQA generates the readout pulses, acquires the down-converted transmitted readout signals for demodulation and discrimination in hardware, and sends the digitized readout results to the HDAWG through the DIO link cable in real time. The HDAWG plays different predefined waveforms conditional on the received readout results from the DIO link cable. The feedback latency, defined as the time interval between sending out the last point of the readout pulse from the UHFQA and sending out the first point of the control pulse from the HDAWG, is about 511 ns in our set-up, which also includes the time for the signal to travel through the experimental circuitry.

### Parity mapping

The parity mapping procedure in the QEC experiment is implemented by applying a classical microwave pulse containing 2*M* (*M* = 11 in our experiment) frequency components on the auxiliary qubit, with the system dynamics governed by the Hamiltonian:

1 $$H/\hslash =-\chi {a}^{\dagger }a|e\rangle \,\langle e|+\Omega \,[\mathop{\sum }\limits_{n\,=\,1}^{2M}{e}^{-i{\delta }_{n}t}|e\rangle \,\langle \,g|+{\rm{h}}\,.{\rm{c}}\,.]$$

in the interaction picture. Here, $\left|e\right\rangle$ denotes the excited state and $\left|g\right\rangle$ denotes the ground state of the auxiliary qubit, *a*^†^ is the creation operator and *a* is the annihilation operator of the photonic field in the cavity, *χ* is the auxiliary qubit’s frequency shift induced per photon as a result of its dispersive coupling, *δ*_*n*_ is the frequency detuning of the *n*-th driving component with a Rabi frequency of Ω and h.c. denotes the Hermitian conjugate. With the choice of the drive frequency detuning *δ*_*n*_ = (2*M* − 2*n* − 1)*χ*, the auxiliary qubit is resonantly driven when the cavity has 2*m* + 1 photons with *m* = 0, 1, …*M*.

For the cavity in the code space, the auxiliary qubit is off-resonantly driven by the comb pulse. For the two-photon state in the cavity, the qubit’s transition $\left|g\right\rangle \leftrightarrow \left|e\right\rangle$ is driven by *M* pairs of frequency components with symmetric detunings, resulting in a qubit state revival at a time of *T* = *k*π/*χ* with *k* being an integer. Similarly, for the zero-photon and four-photon states in the cavity, the qubit is driven by *M* − 1 pairs of symmetric components and two unpaired components, whose effects can be ignored under the condition of 2*Mχ* ≫ Ω. Therefore, the auxiliary qubit also makes a cyclic evolution at *T* = *k*π/*χ* and returns to the initial ground state when the cavity is in the code space.

For the cavity in the error space with one-photon and three-photon states, the auxiliary qubit’s transition $\left|g\right\rangle \leftrightarrow \left|e\right\rangle$ is driven by a resonant frequency component, *M* − 1 pairs of symmetric frequency components and an unpaired off-resonant component. Under the same condition of 2*Mχ* ≫ Ω, we can neglect the off-resonant effect of the unpaired components, and the auxiliary qubit will evolve from the initial ground state to the excited state at *T* = *k*π/*χ*, with *k* being an integer when choosing the drive amplitude Ω = π/2*T*. In our experiment, Ω = *χ*/4, and *T* ≈ π/*χ* for an optimized parity mapping time (see section II in the Supplementary Information).

Therefore, this frequency comb pulse achieves error syndrome detection by mapping the even parity of the cavity state to the auxiliary qubit $\left|g\right\rangle$ state (and the odd parity to the $\left|e\right\rangle$ state) in a quantum non-demolition manner. This parity mapping process can be intuitively illustrated by simultaneously applying two conditional π rotations to the auxiliary qubit to flip the qubit state to the excited state associated with the cavity’s one-photon and three-photon states, thus resulting in a minimum perturbation to the cavity states in the code space.

### Strategies for system optimization

The PASS method^35^ is adopted to mitigate the photon-loss-induced dephasing effect of the logical code words, due to the non-commutativity of the annihilation operation and the self-Kerr term. In our experiment, we apply an off-resonant drive pulse with a frequency detuning of about −3.5*χ* on the auxiliary qubit during the idle operation, resulting in different phase accumulation rates *f*_*n*_ for Fock state $\left|n\right\rangle$ with *n* = 1, 2, 3, 4 relative to the vacuum state. By choosing an optimal amplitude of the detuned drive, we could achieve the error-transparent condition^35^ of (*f*_4_ − *f*_2_) − (*f*_3_ − *f*_1_) = 0 to mitigate the dephasing effect of the logical qubit (Supplementary Fig. 4).

To balance the operation errors, the no-parity-jump backaction errors and the photon-loss errors, we use a two-layer QEC procedure^17^ to improve the QEC performance (see Fig. S6 in the Supplementary Information). In our QEC experiment, there are two bottom layers in a single QEC cycle: the first layer conserves the photon number parity in the deformed code space and the second layer recovers the quantum information in the code space.

The waiting time of the idle operation in each QEC cycle is selected on the basis of a trade-off between the uncorrected errors occurring during this time and the operation errors occurring during the error syndrome measurements and recovery operations. On the one hand, the longer the waiting time, the larger the probability of the two-photon-loss event occurring during this time, which cannot be detected by the lowest-order binomial code. On the other hand, the more frequent the error detection, the more likely it is that the photon-loss errors occur during the detections and corrections. We calculate the QEC lifetime as a function of the waiting time from numerical simulations and choose an optimal waiting time of about 90 μs in our QEC experiment (Supplementary Fig. 8).

## Online content

Any methods, additional references, Nature Portfolio reporting summaries, source data, extended data, supplementary information, acknowledgements, peer review information; details of author contributions and competing interests; and statements of data and code availability are available at 10.1038/s41586-023-05784-4.

## Supplementary information

Supplementary Information This file contains the following four sections and additional references: I. Experimental method; II. Frequency comb control method; III. Details of the QEC procedure; and IV. Error analysis.

## Data Availability

Source data for Figs. 2 and 3 are available with the paper. All other data relevant to this study are available from the corresponding author upon reasonable request.

## Source data

Source Data Fig. 2
Source Data Fig. 3

## Code availability

The code used for simulations is available from the corresponding author upon reasonable request.
