# Supplementary material for: Beating the break-even point with a discrete-variable-encoded logical qubit
Source: Nature. 2023 Mar 22;616(7955):56–60. doi: 10.1038/s41586-023-05784-4 (PMC10076216; doi:10.1038/s41586-023-05784-4)
Supplement: Supplementary file 1 — This file contains the following four sections and additional references: I. Experimental method; II. Frequency comb control method; III. Details of the QEC procedure; and IV. Error analysis. [file 41586_2023_5784_MOESM1_ESM.pdf]

nature portfolio

https://doi.org/10.1038/s41586-023-05784-4

Supplementary information

Beating the break-even point with a discrete-variable-encoded logical qubit

In the format provided by the authors and unedited

Supplementary Information for "Beating the break-even point with a discrete-variable-encoded logical qubit"

Zhongchu Ni,[1,2,3] Sai Li,[1,2] Xiaowei Deng,[1,2] Yanyan Cai,[1,2] Libo Zhang,[1,2] Weiting Wang,[4] Zhen-Biao Yang,[5] Haifeng Yu,[6] Fei Yan,[1,2] Song Liu,[1,7,2] Chang-Ling Zou,[8,9] Luyan Sun,[4,9,*] Shi-Biao Zheng,[5,†] Yuan Xu,[1,7,2,‡] and Dapeng Yu[1,7,2,3,§]

[1]*Shenzhen Institute for Quantum Science and Engineering, Southern University of Science and Technology, Shenzhen 518055, China*
[2]*Guangdong Provincial Key Laboratory of Quantum Science and Engineering, Southern University of Science and Technology, Shenzhen 518055, China*
[3]*Department of Physics, Southern University of Science and Technology, Shenzhen 518055, China*
[4]*Center for Quantum Information, Institute for Interdisciplinary Information Sciences, Tsinghua University, Beijing 100084, China*
[5]*Fujian Key Laboratory of Quantum Information and Quantum Optics, College of Physics and Information Engineering, Fuzhou University, Fuzhou, Fujian 350108, China*
[6]*Beijing Academy of Quantum Information Sciences, Beijing 100193, China*
[7]*International Quantum Academy, and Shenzhen Branch, Hefei National Laboratory, Futian District, Shenzhen 518048, China*
[8]*CAS Key Laboratory of Quantum Information, University of Science and Technology of China, Hefei, Anhui 230026, China*
[9]*Hefei National Laboratory, Hefei 230088, China*

I. EXPERIMENTAL METHOD

A. Device

The quantum error correction (QEC) experiment is implemented in a three-dimensional (3D) circuit quantum electrodynamics (QED) architecture [1–6], which consists of a superconducting transmon qubit [7], a 3D coaxial stub cavity [8–10], and a Purcell-filtered stripline readout resonator [11, 12]. A schematic of the device is shown in Fig. S1. The 3D circuit QED device is directly machined from a single block of high purity (5N5) aluminum and chemically etched to improve the cavity's coherence time [13].

The coaxial stub cavity is constructed as a 3D $\lambda/4$ transmission line resonator with the fundamental mode used for storing microwave photons and encoding the bosonic logical qubit, henceforth referred to as the storage cavity. The Purcell-filtered readout resonator is constructed with two quasi-planar $\lambda/2$ transmission line resonators, which are formed by the metal wall of a horizontal tunnel and two metal strips on the qubit chip inserted in the tunnel. The transmon qubit is patterned on a sapphire chip with two antenna pads to couple to the storage cavity mode and the stripline readout resonator mode. The Josephson junction of the qubit is an $\mathrm{Al}-\mathrm{Al_2O_3}-\mathrm{Al}$ trilayer tunnel junction formed by a double angle evaporation technique, and the antenna pads and readout striplines are grown using tantalum films in BCC alpha-phase to improve the coherence time of the transmon qubit [14, 15].

In our experiment, the transmon qubit serves as an auxiliary qubit for error detection and correction operations of the bosonic logical qubit in the storage cavity. The stripline readout resonator is strongly coupled to the transmon qubit for fast dispersive readout of the qubit states, and coupled to the outside world via another quasi-planar $\lambda/2$ transmission line resonator, denoted as the Purcell filter resonator, to protect the coherence times of both the auxiliary qubit and the storage cavity. To fit both the readout resonator and filter resonator, the striplines are designed with wiggles to decrease the physical footprint of the patterns on the sapphire chip [12].

*Electronic address: luyansun@tsinghua.edu.cn
†Electronic address: t96034@fzu.edu.cn
‡Electronic address: xuy5@sustech.edu.cn
§Electronic address: yudp@sustech.edu.cn

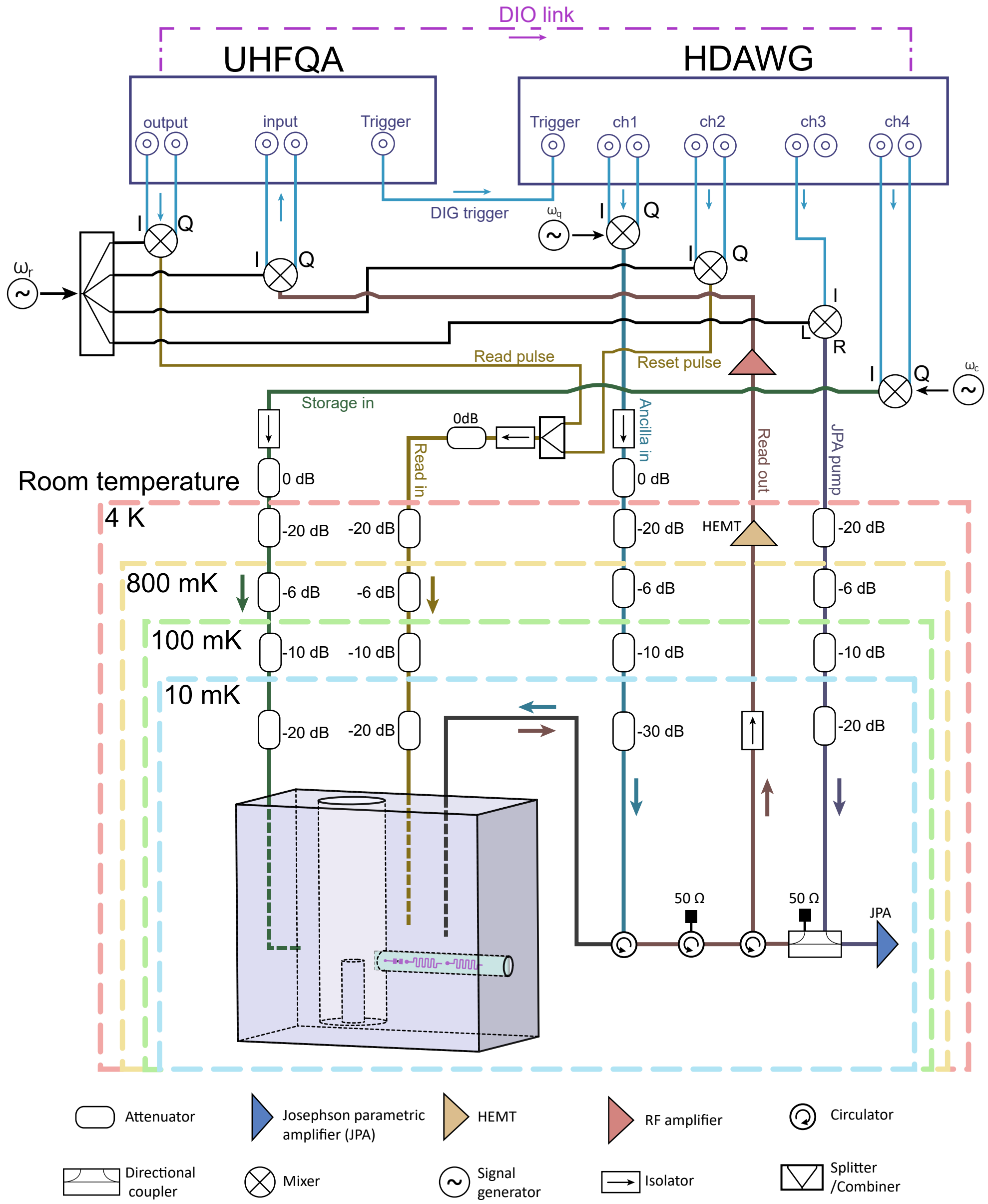

FIG. S1: Full wiring of the experimental circuitry and device schematic.

B. Setup

The experimental device is covered with a magnetic shield and installed inside a cryogenic-free dilution refrigerator, which has a temperature below 10 mK. Both the qubit and bosonic logical qubit in the storage cavity are controlled with microwave pulses generated by single-sideband in-phase and quadrature (IQ) modulations. The corresponding waveforms for each mode are generated from two digital-to-analog converter (DAC) channels of the Zurich Instruments high-density arbitrary waveform generator (ZI HDAWG). The qubit control pulses have a cosine-shaped envelope with a duration of about 20 ns, in combination with the technique of "derivative removal by adiabatic gate" (DRAG) to remove leakage errors to higher energy levels [16, 17]. The cavity control pulses for encoding and decoding the bosonic logical qubit and implementing the recovery operations after each error detection are generated from numerical optimization with the gradient ascent pulse engineering (GRAPE) method [18]. The readout pulse is generated by single-sideband IQ modulation of the waveforms from two DAC channels of a Zurich Instruments ultra-high frequency quantum analyzer (ZI UHFQA) in cooperation with a signal generator as the local oscillator and sent to the readout

TABLE S1: Hamiltonian parameters and coherence times.

Measured (predicted) parameters		Transmon qubit	Storage cavity	Readout resonator
Mode frequency $\omega_{\mathrm{q,c,r}}/2\pi$		4.962 GHz	6.532 GHz	8.562 GHz
Kerr interactions	Transmon qubit	216 MHz	2.59 MHz	1.9 MHz
	Storage cavity	2.59 MHz	9.7 kHz	(12.7 kHz)
	Readout resonator	1.9 MHz	(12.7 kHz)	(4.2 kHz)
Higher-order self-Kerr $K'_{\mathrm{c}}/2\pi$			0.32 kHz	
Higher-order cross-Kerr $\chi'_{\mathrm{qc}}/2\pi$		5.41 kHz		
Relaxation time T_1		98 μs	578 μs	58 ns
Pure dephasing time T_ϕ		968 μs	4389 μs	-
Thermal population		1.3%	0.6%	$< 0.1\%$

resonator through a coaxial cable with a series of attenuators and filters.

The transmitted signal from the resonator is amplified by a series amplification chain, including a quantum limited Josephson parametric amplifier (JPA) at the base temperature, a high electron mobility transistor (HEMT) at the 4K stage, and a standard commercial low-noise RF amplifier at room temperature. Finally, the signal is downconverted by an IQ mixer with the same local oscillator as that used to generate the readout pulse. The downconverted IQ signals are digitized and recorded by the analogy-to-digital converter (ADC) of the same ZI UHFQA. To align the timing sequence of the qubit and cavity control pulses with the readout pulse, the UHFQA serves as a master instrument to send a trigger signal to control the slave instrument HDAWG. Figure S1 shows a schematic of the full wiring of the experimental setup.

The QEC experiment requires both error syndrome measurements and error corrections in real time, which is realized by connecting the UHFQA to the HDAWG with a digital input/output (DIO) link cable. The UHFQA has the capabilities of performing the demodulation and calculations of the downconverted readout signals in hardware and discriminating the results into digitized signals in real time. These digital signals are not only sent to the host PC, but also to the HDAWG for fast feedback control through the DIO link. The HDAWG can store a series of predefined waveforms and selectively play waveforms conditional on the received DIO signal in real time, which realizes the quantum feedback control for quantum error correction operations with a minimized latency. The duration of sending the last point of the readout pulse from UHFQA and sending out the first point of the feedback control signal is defined as the feedback latency, which is about 511 ns in our experiment (including the signal travelling time through the experimental circuitry).

C. System Hamiltonian

The Hamiltonian of our system containing a storage cavity, a readout resonator, and an auxiliary qubit can be expressed in the dispersive regime as

$$\begin{aligned} \hat{H}/\hbar &= \omega_{\mathrm{q}}\hat{a}_{\mathrm{q}}^\dagger\hat{a}_{\mathrm{q}} + \omega_{\mathrm{c}}\hat{a}_{\mathrm{c}}^\dagger\hat{a}_{\mathrm{c}} + \omega_{\mathrm{r}}\hat{a}_{\mathrm{r}}^\dagger\hat{a}_{\mathrm{r}} \\ &- \frac{K_{\mathrm{q}}}{2}\hat{a}_{\mathrm{q}}^{\dagger 2}\hat{a}_{\mathrm{q}}^2 - \frac{K_{\mathrm{c}}}{2}\hat{a}_{\mathrm{c}}^{\dagger 2}\hat{a}_{\mathrm{c}}^2 + \frac{K'_{\mathrm{c}}}{6}\hat{a}_{\mathrm{c}}^{\dagger 3}\hat{a}_{\mathrm{c}}^3 - \frac{K_{\mathrm{r}}}{2}\hat{a}_{\mathrm{r}}^{\dagger 2}\hat{a}_{\mathrm{r}}^2 \\ &- \chi_{\mathrm{qr}}\hat{a}_{\mathrm{q}}^\dagger\hat{a}_{\mathrm{q}}\hat{a}_{\mathrm{r}}^\dagger\hat{a}_{\mathrm{r}} - \chi_{\mathrm{cr}}\hat{a}_{\mathrm{c}}^\dagger\hat{a}_{\mathrm{c}}\hat{a}_{\mathrm{r}}^\dagger\hat{a}_{\mathrm{r}} - \chi_{\mathrm{qc}}\hat{a}_{\mathrm{q}}^\dagger\hat{a}_{\mathrm{q}}\hat{a}_{\mathrm{c}}^\dagger\hat{a}_{\mathrm{c}} + \frac{\chi'_{\mathrm{qc}}}{2}\hat{a}_{\mathrm{q}}^\dagger\hat{a}_{\mathrm{q}}\hat{a}_{\mathrm{c}}^{\dagger 2}\hat{a}_{\mathrm{c}}^2, \end{aligned} \tag{S1}$$

where $\omega_{\mathrm{q,c,r}}$ are the frequencies of the auxiliary qubit, storage cavity, and readout resonator, respectively; $\hat{a}_{\mathrm{q,c,r}}(\hat{a}_{\mathrm{q,c,r}}^\dagger)$ are their corresponding annihilation (creation) operators; $K_{\mathrm{q,c,s}}$ are the self-Kerrs of the corresponding mode; K'_{c} is a higher-order self-Kerr of the storage cavity; χ_{qr}, χ_{cr}, and χ_{qc} are the cross-Kerrs between these three modes; and χ'_{qc} is the higher-order cross-Kerr between the auxiliary qubit and the storage cavity. All these parameters are summarized and listed in Table S1. Note that some parameters cannot be directly measured and are predicted according to $\chi_{ab} = 2\sqrt{K_a K_b}$ based on black-box quantization (BBQ) theory [19].

When only considering the transmon qubit in the two lowest energy levels and discarding the readout resonator mode during the parity mapping process in the QEC experiment, the above Hamiltonian can be rewritten as

$$\hat{H}_{\mathrm{qc}}/\hbar = (\omega_{\mathrm{q}} - \chi_{\mathrm{qc}}\hat{a}_{\mathrm{c}}^\dagger\hat{a}_{\mathrm{c}})\,|e\rangle\langle e| + \omega_{\mathrm{c}}\hat{a}_{\mathrm{c}}^\dagger\hat{a}_{\mathrm{c}} - \frac{K_{\mathrm{c}}}{2}\hat{a}_{\mathrm{c}}^{\dagger 2}\hat{a}_{\mathrm{c}}^2, \tag{S2}$$

where $|e\rangle$ ($|g\rangle$) denotes the auxiliary qubit excited (ground) state. With this dispersive interaction between the auxiliary qubit and the storage cavity, the qubit transition frequency is modified by the photon number in the cavity with an energy level spacing of χ_{qc} per photon. This homogeneous feature allows for implementing the parity mapping procedure with the frequency comb control method, which will be discussed in detail in Sec. II.

D. Coherence times and readout fidelities

The coherence times and thermal populations of the qubit and cavity modes are experimentally measured and are also summarized in Table S1. The auxiliary qubit has an average energy relaxation time of $T_1 = 98$ μs and a Ramsey coherence time of $T_2^* = 163$ μs, inferring a pure dephasing time of $T_\phi = 968$ μs, while the storage cavity has a single-photon lifetime of $T_1 = 578$ μs ($\kappa_c/2\pi = 0.28$ kHz) and a Ramsey coherence time of $T_2^* = 915$ μs, corresponding to a pure dephasing time of $T_\phi = 4389$ μs. The readout resonator is designed to have an energy relaxation time of about 58 ns ($\kappa_r/2\pi = 2.7$ MHz) for fast single-shot qubit readout. The pure dephasing time of the storage cavity is about seven times larger than the single-photon energy relaxation time, indicating that the photon-loss error is the dominant error source for the logical qubit. The thermal populations of the auxiliary qubit and the storage cavity are $n_{\mathrm{th}}^{\mathrm{q}} = 1.3\%$ and $n_{\mathrm{th}}^{\mathrm{c}} = 0.6\%$, respectively. The thermal population of the readout resonator $n_{\mathrm{th}}^{\mathrm{r}}$ is inferred by the qubit pure dephasing time $T_\phi < 1/n_{\mathrm{th}}^{\mathrm{r}}\kappa_r$ [20], giving an upper bound of the readout resonator thermal population of $n_{\mathrm{th}}^{\mathrm{r}} < 0.1\%$.

The dispersive interaction between the auxiliary qubit and the readout resonator allows for quantum non-demolition (QND) measurement of the qubit states, which is achieved by measuring the transmission signals through the readout resonator. The decay rate of the readout resonator $\kappa_r/2\pi = 2.7$ MHz is designed to match the dispersive coupling strength $\chi_{\mathrm{qr}}/2\pi = 1.9$ MHz between the qubit and readout resonator to improve the signal-to-noise ratio of the readout signals and achieve the high-fidelity (with the help of a JPA) and high-QND single-shot readout of the auxiliary qubit. The readout pulse has an optimized duration of about 600 ns, giving an average readout fidelity of 0.993 (0.998 for the $|g\rangle$ state and 0.988 for the $|e\rangle$ state), and an average QNDness of 0.985 (0.998 for the $|g\rangle$ state and 0.972 for the $|e\rangle$ state).

E. Quantum optimal control

The control pulses of the storage cavity for encoding and decoding the bosonic logical qubit and implementing the recovery operations are generated from quantum optimal control with GRAPE technique [18]. Each ideal unitary operation U_{ideal} is implemented by applying two control pulses $\epsilon_q(t)$ and $\epsilon_c(t)$ to drive both the auxiliary qubit and the cavity, in order to realize a set of simultaneous state transfers to map each initial state $|\psi_0^i\rangle$ to the corresponding final state $|\psi_f^i\rangle = U_{\mathrm{ideal}}|\psi_0^i\rangle$ for the i-th initial state in the relevant subspace. The goal for the quantum control is to maximize the average fidelity $F = \left|\sum_i \langle\psi_f^i|U(\epsilon_q(t),\epsilon_c(t))|\psi_0^i\rangle\right|^2$ of these state transfers by optimizing the control pulses $\epsilon_q(t)$ and $\epsilon_c(t)$. Here, $U(\epsilon_q(t),\epsilon_c(t))$ is the unitary evolution operator with the corresponding drive pulses applied on the system.

In order to numerically solve this optimization problem, we assume these control pulses are piecewise constant functions by dividing the total gate time T into N segments with each duration of $\Delta t = T/N$. Then the total evolution operator U can be expressed as $U(\epsilon_q(t),\epsilon_c(t)) = U_N U_{N-1} ... U_2 U_1$, where $U_k = \exp\left(-i(H_0 + H_d)\Delta t/\hbar\right)$ represents the evolution operator of the k-th segment. Here H_0 is the drift Hamiltonian, describing the dispersive interaction between the auxiliary qubit and the storage cavity, $H_d = \epsilon_q^I(k\Delta t)\sigma_x + \epsilon_q^Q(k\Delta t)\sigma_y + \epsilon_c^I(k\Delta t)(\hat{a}_{\mathrm{c}} + \hat{a}_{\mathrm{c}}^\dagger) + i\epsilon_c^Q(k\Delta t)(\hat{a}_{\mathrm{c}} - \hat{a}_{\mathrm{c}}^\dagger)$ is the driving Hamiltonian for both the auxiliary qubit and the cavity at the k-th segment, and ϵ^I and ϵ^Q are the in-phase and quadrature components of the two drives.

By analytically calculating the gradient of the fidelity with respect to all the control fields, the optimization problem can be efficiently solved by directly using the quasi-Newton optimization algorithms. In practice, we also add some additional penalty terms to the optimization cost function, in order to make the resulting solution robust to experimental imperfections.

II. FREQUENCY COMB CONTROL METHOD

A. Theory

In the QEC experiment with a binomially encoded logical qubit in the storage cavity, the error syndrome is measured by mapping the photon number parity of the cavity state to the auxiliary qubit state. This is achieved by applying a classical microwave pulse containing $2M$ frequency components on the auxiliary qubit. In the interaction picture, the system dynamics are governed by the Hamiltonian:

$$\hat{H}_I = -\chi_{\mathrm{qc}}\hat{a}_{\mathrm{c}}^\dagger\hat{a}_{\mathrm{c}} \left|e\right\rangle\left\langle e\right| + \sum_{n=1}^{2M}\Omega_n e^{-i\delta_n t}\left|e\right\rangle\left\langle g\right| + h.c., \tag{S3}$$

where Ω_n and δ_n are the drive amplitude and frequency detuning of the n-th driving component. For simplicity, χ_{qc} will be denoted as χ in the following description. With the choice of $\delta_n = (2M-2n-1)\chi$ and $\Omega_n = \Omega$, each of the transitions of $\left|g, 2m+1\right\rangle \leftrightarrow \left|e, 2m+1\right\rangle$ with $m = 0, 1, 2...M$ is resonantly driven by a microwave frequency component, and off-resonantly driven by $2M-1$ components with detunings of $2k\chi$ with $k = -(M-m)$ to $M+m-1$ and $k \neq 0$. On the other hand, each of the transitions $\left|g, 2m\right\rangle \leftrightarrow \left|e, 2m\right\rangle$ is also off-resonantly driven by $2M$ frequency components with detunings of $(2k-1)\chi$ with $k = -(M-m)$ to $M+m-1$.

For the cavity in the code space with a 2-photon state, the transition $\left|g, 2\right\rangle \leftrightarrow \left|e, 2\right\rangle$ is driven by M pairs of frequency components with detunings $\pm\chi, \pm3\chi, ... \pm(2M-1)\chi$. After a pulse duration of T, the initial state $\left|g, 2\right\rangle$ evolves to

$$\cos\xi\left|g, 2\right\rangle - ie^{2i\chi_{qc}T}\sin\xi\left|e, 2\right\rangle, \tag{S4}$$

where

$$\xi = \sum_{n=0}^{M-1} 2\int_0^T dt\Omega\cos\left[(2n+1)\chi t\right]. \tag{S5}$$

For Ω being a constant, we have $\xi = 2\sum_{n=0}^{M-1}\frac{\Omega}{(2n+1)\chi}\sin\left[(2n+1)\chi T\right]$. With the choice of $\chi T = m\pi$ ($m = 1, 2, 3, ...$ is an integer), the qubit finally returns to the ground state and nothing changes.

For the cavity in the code space with 0- and 4-photon states, the auxiliary qubit's $\left|g\right\rangle \leftrightarrow \left|e\right\rangle$ transition is driven by $M-1$ pairs of frequency components with detunings of $\pm\chi, \pm3\chi, ..., \pm(2M-3)\chi$ and two unpaired frequency components with detunings of $(2M\pm1)\chi$. Supposing that $(2M-1)\chi \gg \Omega$, the effect of the two unpaired frequency components can be neglected. After the pulse duration T, the initial states $\left|g, 0\right\rangle$ and $\left|g, 4\right\rangle$ make a cyclic evolution and return to the original states.

For the cavity in the error space with 1- and 3-photon states, the auxiliary qubit's transition $\left|g\right\rangle \leftrightarrow \left|e\right\rangle$ is driven by $M-1$ pairs of frequency components with detunings of $\pm2\chi, \pm4\chi, ... \pm2(M-1)\chi$, one resonant frequency component, and one unpaired frequency component with detuning of $2M\chi$. When the effect of the unpaired drive is neglected under the condition of $2M\chi \gg \Omega$, the system's evolution is

$$\cos\mu\left|g, k\right\rangle - ie^{ik\chi T}\sin\mu\left|e, k\right\rangle, k = 1, 3, \tag{S6}$$

where

$$\mu = \Omega T + 2\sum_{n=1}^{M-1}\frac{\Omega}{2n\chi}\sin(2n\chi T). \tag{S7}$$

With the choice of $\Omega T = \pi/2$ and $\chi T = m\pi$ with $m = 1, 2, 3, ...$ being an integer, both the $\left|g, 1\right\rangle$ and $\left|g, 3\right\rangle$ states are transformed to $\left|e, 1\right\rangle$ and $\left|e, 3\right\rangle$, respectively. Therefore, the detection of the auxiliary qubit in state $\left|g\right\rangle$ indicates that no photon loss has occurred, and the logical qubit remains in the code space with even parity. The detection of the auxiliary qubit in $\left|e\right\rangle$ indicates that a single-photon-loss error has occurred and the cavity is in the error space with odd parity.

B. Frequency comb pulse optimization

According to the theory described above, all frequency components are assumed to have identical driving amplitudes, which are chosen to be $\Omega = \chi/4$ in our experiment for the parity mapping. However, the auxiliary qubit

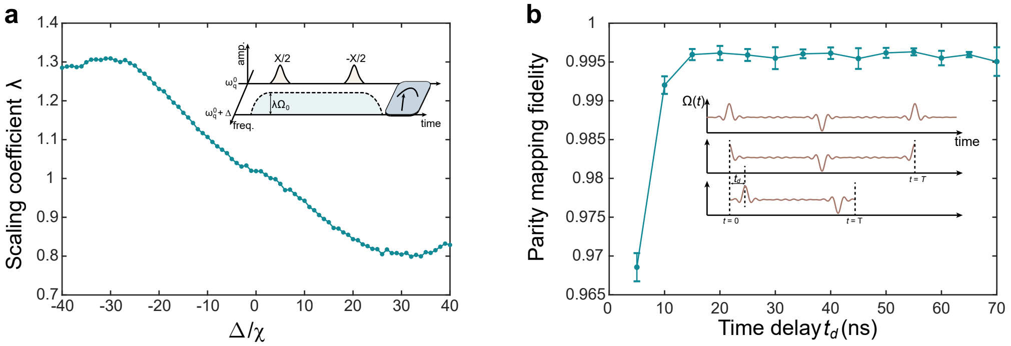

FIG. S2: Calibration and optimization of the frequency comb pulse. (a) Calibration of the scaling coefficient of the pulse amplitude for each frequency component. (b) The measured parity detection fidelity as a function of the delay time of the frequency comb pulse envelope in order to suppress the pulse distortion.

may have different responses for each frequency component drive with different frequency detunings due to the frequency-dependent transmission characteristic of the microwave control circuit. Thus, to achieve high-fidelity parity measurement, the pulse amplitude of each frequency component should be carefully calibrated.

The drive amplitude of each frequency component is calibrated by performing a Ramsey experiment and measuring the a.c. Stark frequency shift. The magnitude of the a.c. Stark shift of the qubit frequency can be expressed as

$$|\delta| = \frac{1}{2}\left(\sqrt{\Delta^2 + (\lambda\Omega_0)^2} - |\Delta|\right), \tag{S8}$$

where Δ is the frequency detuning of the microwave tone, Ω_0 is the uncalibrated drive strength of the detuned pulse, and λ is the amplitude scaling coefficient. In our experiment, we calibrate the amplitude scaling by applying an off-resonant drive to the auxiliary qubit with frequency detunings of $\Delta = n\chi$ $(n = \pm1, \pm2, ...)$ and measuring the corresponding frequency shift from the Ramsey experiment. The calibration results are shown in Fig. S2a.

In our experiment, the frequency comb pulse is generated by mixing the multi-frequency pulses with a single local oscillator, whose frequency is aligned to the qubit frequency with two photons in the cavity. Thus, the envelope of the frequency comb waveforms can be expressed as

$$\Omega_{\mathrm{comb}}(t) = \Omega\sum_{n=1}^{M}\{\cos[(2n-1)\chi(t-t_d)] + \cos[-(2n-1)\chi(t-t_d)]\}, \quad t \in [0, T], \tag{S9}$$

where t_d is the delay time to introduce a phase shift for each frequency component. With $t_d = 0$, the comb pulse envelope has a large amplitude at the initial and final time for the parity mapping, which will inevitably result in pulse distortions and reduce the control fidelity in the actual experiment.

In our experiment, we introduce a phase shift for each frequency component by adding a delay time for the comb pulse envelope, thus making the pulse amplitudes approach zero at the initial and final time due to the destructive interference of all components. In the meantime, the total length of the parity mapping procedure can also be reduced by a factor of 2, approaching a time of about π/χ when choosing the drive strength $\Omega = \chi/4$. Therefore, the pulse duration for comb parity mapping is similar to that in the Ramsey interferometer but with a smaller pulse amplitude. In our experiment, we measure the parity fidelity as a function of the delay time, with the experimental result shown in Fig. S2b. An optimal value of the delay time is chosen as 47 ns in our experiment to make the pulse amplitudes sufficiently small at the initial and final time. In addition, 5-ns rising and falling edges are also added to further smooth the waveforms of the frequency comb pulses. As a result, the optimal comb driving pulse for the parity measurement has a total length of 255 ns with 22 frequency components in the QEC experiment.

C. Parity measurement fidelity

We first characterize the parity measurement fidelity by directly measuring the photon number parity of the vacuum state $|0\rangle$, giving a fidelity of 0.994. In addition, we also measure the photon number parities of even and odd cat states with different average photon numbers, which are generated by performing a parity measurement on an initial coherent state, post-selecting the parity measurement result, and performing another two consecutive parity measurements. The first two consecutive identical parity results would give a photon state parity with good confidence, and are

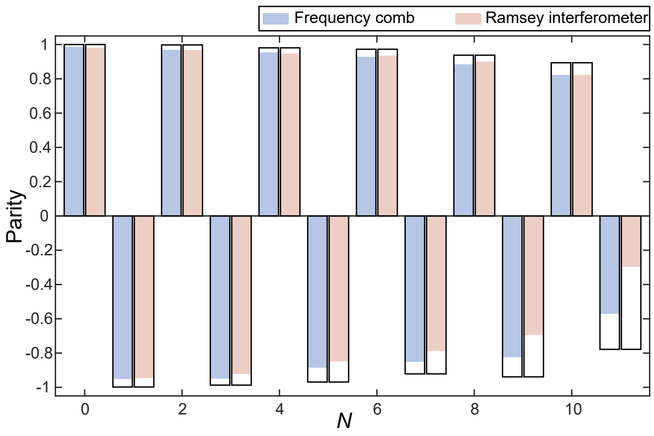

FIG. S3: Bar chats of the measured parities for different photon number states $|N\rangle$ with both the frequency comb method and the Ramsey interferometer method. Solid black frames represent the ideal photon number parities from numerical simulations.

post-selected to estimate the parity fidelity from the third parity measurement. The experiment gives an average parity fidelity of 0.987 for $\bar{n} = 1$, 0.985 for $\bar{n} = 2$, and 0.976 for $\bar{n} = 3$.

For the logical qubit encoded with the lowest-order binomial code, the parity measurement fidelity for the cavity states in the code and error spaces are also measured in a similar manner. In our experiment, we first encode the cavity into each cardinal-point state in the code and error spaces, and then perform three consecutive parity measurements. Post-selection of the first two identical parity results would give better confidence for estimating the parity measurement fidelity from the third parity measurement. The experimental results are shown in Fig. 2 in the main text, indicating an average parity detection fidelity of 0.989 and 0.975 for the cavity states in the code and error spaces, respectively.

D. Comparison with the Ramsey method

The conventional photon number parity detection of the cavity states is implemented by a Ramsey interferometer, where a qubit-state-dependent π-phase shift of the cavity is sandwiched between two unconditional $\pi/2$ pulses applied on the auxiliary qubit. During the parity mapping, the auxiliary qubit evolves in the equatorial plane of the qubit Bloch sphere most of the time and has an average excited state population of 0.5, no matter whether the cavity is in the code or error spaces. A qubit relaxation error during the parity mapping will give a wrong indication of the following correction operation, resulting in a depolarization error of the logical qubit. Meanwhile, the auxiliary qubit also suffers largely from dephasing noise during parity mapping with this type of parity measurement. In addition, the Ramsey interferometer necessitates unconditional $\pi/2$ pulses, which cannot be perfectly achieved for the multiphoton encoded logical states due to the photon-number-dependent dispersive shift of the auxiliary qubit frequency. Furthermore, it will generally deteriorate for large photon number encodings in the cavity, due to the inevitable introduction of off-resonant driving errors.

As a distinct contrast, the ingenious designed frequency comb method for the parity mapping procedure in QEC can mitigate these adverse effects to some extent. Compared to the Ramsey method, the auxiliary qubit would have a small excited state population (less than 0.5) during the frequency comb parity mapping procedure for the cavity in code space with 0-, 2- and 4-photon states with carefully designed frequency comb parameters. In a QEC experiment, there is only a small probability of the single-photon-loss error occurring and the cavity still remains in the code space with a large proportion. Therefore, reducing the auxiliary qubit excited state population when the cavity is in the even parity subspace will suppress the auxiliary qubit relaxation errors during the parity mapping and thus finally benefit the whole QEC process. Besides, the continuous driving comb effectively decouples the auxiliary qubit from dephasing noises during the error syndrome mapping [21].

In addition, this frequency comb method also allows for measuring the parity of large photon number states because of no need for unconditional qubit rotations. In our experiment, we directly compare the performance of the frequency comb method with that of the conventional Ramsey method to measure the parities of large photon number states. In Fig. S3, we present the measured photon number parities with these two methods for various initial Fock states $|N\rangle$, which are generated by using the numerical optimization method. The experimental results indicate that the comb method indeed has an obvious benefit for large photon number states.

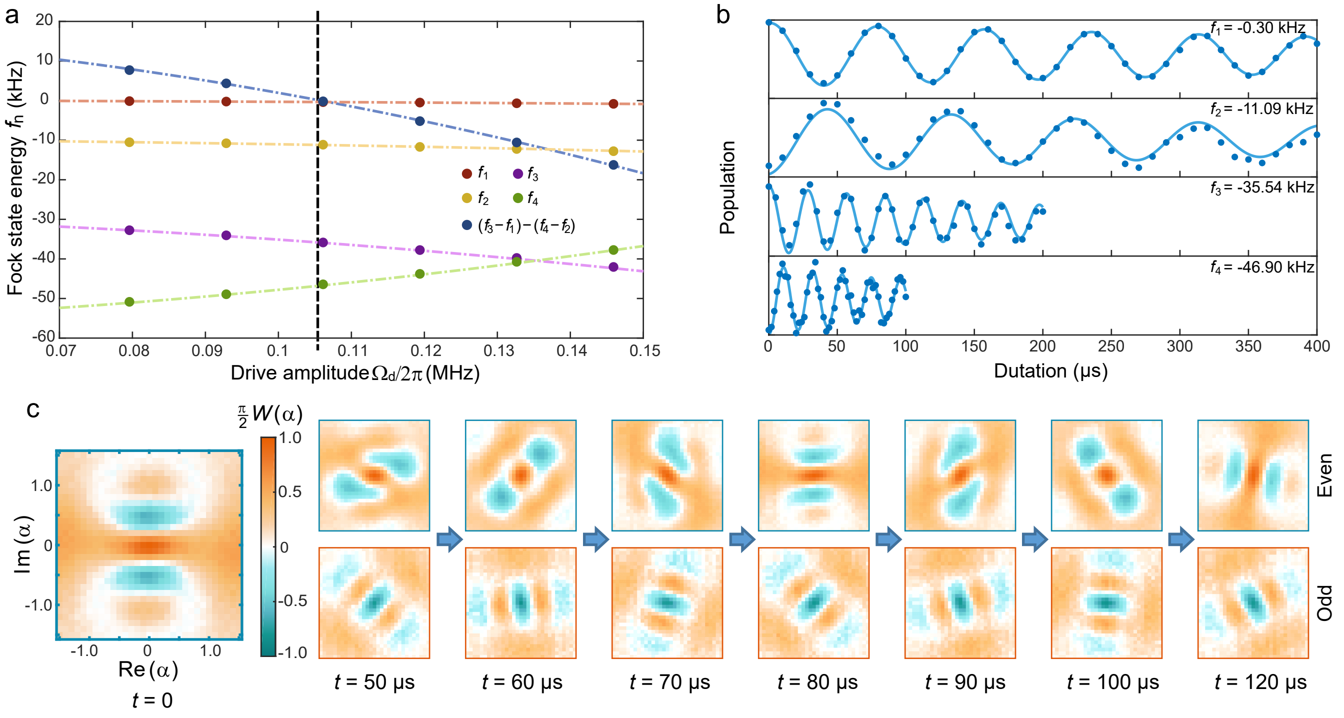

FIG. S4: Eliminating the dephasing effect with PASS drive. (a) Measured phase accumulation rates f_n for Fock states $|n\rangle$ (with $n = 1, 2, 3, 4$) relative to $|0\rangle$, as well as the difference between them $\Delta f = (f_3 - f_1) - (f_4 - f_2)$. (b) Measured Ramsey oscillations between Fock state $|n\rangle$ (with $n = 1, 2, 3, 4$) and $|0\rangle$ at the optimal amplitude of the PASS drive. (c) Measured Wigner functions of the cavity states after different evolution times conditional on the parity measurement results.

As another distinct advantage, the frequency comb control method can also be generalized to perform error syndrome detection of higher-order binomial codes, which can be used to detect and correct the errors for more than one photon losses [22]. The basis states of these codewords have different photon number parities, with the generalized photon number parity serving as the error syndrome, which cannot be directly measured with the conventional Ramsey method. In contrast, the high tailorability of the frequency comb method does not require that the two basis states of the codeword have the same parity and can be easily adapted to the higher-order binomial codes.

III. DETAILS OF THE QEC PROCEDURE

The QEC experiment starts by initializing both the auxiliary qubit and the storage cavity in the ground state $|g, 0\rangle$, which is achieved by post-selections of the auxiliary qubit's ground state and a subsequent cavity parity measurement since both the auxiliary qubit and the storage cavity have small thermal excitations.

After the initialization, the auxiliary qubit is first prepared to the six cardinal-point states in the qubit Bloch sphere, and transferred to the binomial logical space $\{|0_L\rangle, |1_L\rangle\}$ of the storage cavity with an encoding process, which is realized by applying a numerically optimized pulse with a duration of about 770 ns.

The quantum information stored in the logical states is protected by repetitive QECs implemented after the encoding operation, and is extracted by decoding back to the auxiliary qubit and performing the tomography experiment on the auxiliary qubit. A single QEC cycle has a total duration of about 92.46 μs containing: a waiting time of $t_w \approx 90$ μs, a frequency comb pulse (with a total width of about 255 ns) to measure the photon number parity, a qubit readout pulse with a duration of about 600 ns, a waiting time of about 511 ns after the measurement to release the readout cavity photons and demodulate and digitize readout signals for feedback control, a 20 ns cosine-shaped unconditional π pulse to reset the auxiliary qubit, and correction operations (GRAPE pulse with a width of about 770 ns) conditional on the previous measurement result.

During the waiting time of t_w in each QEC cycle, an off-resonant drive pulse with smooth rising and falling edges (100 ns for each) is applied on the auxiliary qubit to mitigate the self-Kerr induced dephasing effect of the logical codewords in the storage cavity by using the photon-number-resolved a.c. Stark shift (PASS) method [23]. In our experiment, we measure the phase accumulation rates f_n for Fock states $|n\rangle$ with $n = 1, 2, 3, 4$ relative to the vacuum

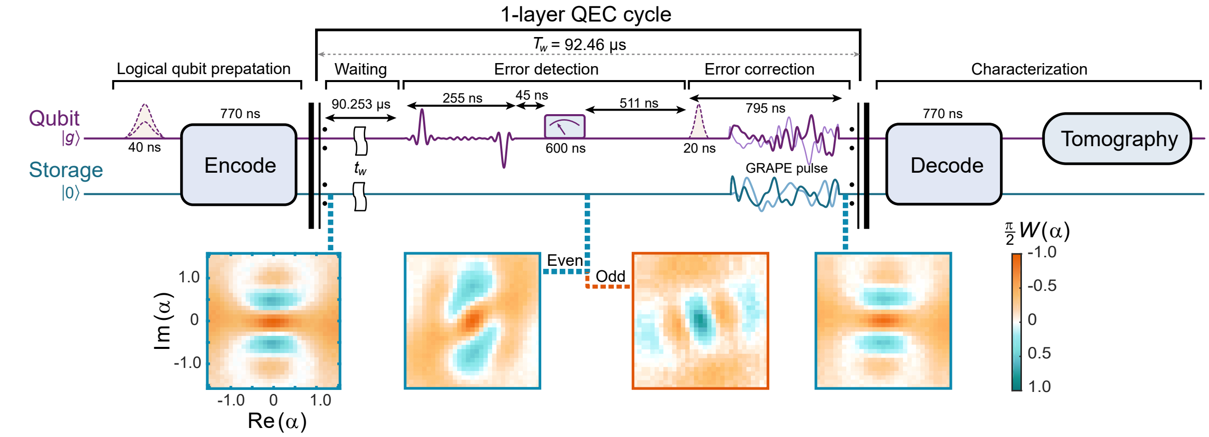

FIG. S5: Experimental sequence for the one-layer QEC experiment, as well as the Wigner snapshots of the cavity state at each time step in the QEC sequence.

state $|0\rangle$ as a function of the drive amplitude but with a fixed frequency detuning of -3.5χ, and present the results in Fig S4a. By adopting an optimal drive amplitude of $\Omega_d/2\pi = 0.106$ MHz, the accumulation rates measured from Ramsey experiments and shown in Fig. S4b, meet the error-transparent condition of $(f_4 - f_2) - (f_3 - f_1) = 0$ to eliminate the dephasing effect of the logical qubit. In order to further check the quantum evolution in the code and error spaces, we measure the Wigner functions of the cavity states by post-selecting the parity measurement result after various evolution time, with the experimental results shown in Fig. S4c. The results indicate that the phase coherence in the error space is significantly preserved, manifesting the tolerance of the stochastic single-photon-jump error during the waiting time.

Figure S5 shows the experimental sequence of the one-layer QEC process, as well as the Wigner snapshots at each time step in the sequence. The feedback latency of the adaptive control is defined as the time interval between sending out the last point of the readout signal and sending out the first point of the qubit control signal, which also includes the travelling time through the whole experimental circuitry, and is about 511 ns in our experiment.

In order to balance the operation errors, no-parity-jump backaction errors, and photon-loss errors, we adapt a two-layer QEC procedure [24] to improve the error correction performance, with the protocol details shown in Fig. S6.

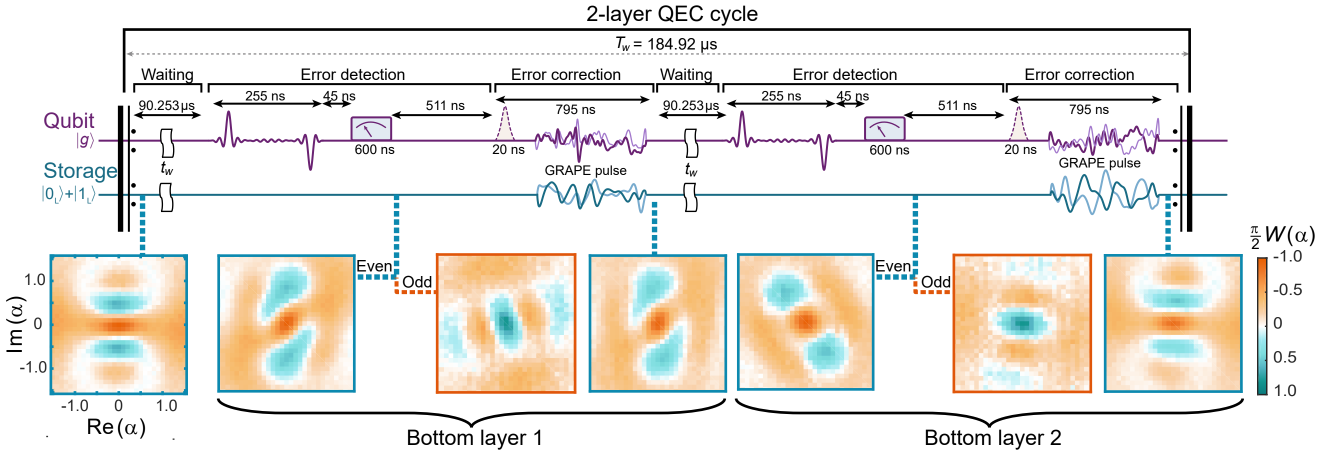

FIG. S6: Experimental sequence for the two-layer QEC experiment, as well as the Wigner snapshots of the cavity state at each time step in the QEC sequence.

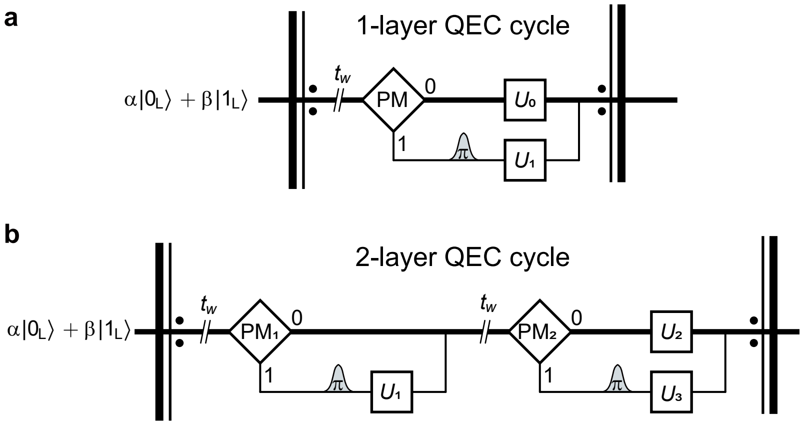

FIG. S7: Schematic of the one-layer (a) and two-layer (b) QEC cycles in the experiment.

IV. ERROR ANALYSIS

In order to understand the experimental results of the QEC performance, we investigate the error sources and their contributions to the loss of fidelity for each QEC cycle in this section. According to an analytical model in Ref. [24], we divide the error sources in the QEC procedure into four parts: the intrinsic error, the parity measurement infidelity, the recovery operation infidelity, and the auxiliary qubit thermal excitation error for both the one- and two-layer QEC experiments, with the schematics shown in Fig. S7. These errors are summarized and listed in Table S2. Detailed descriptions for estimating and calculating these errors are presented below. Note that the process fidelity has a minimum of 0.25, and thus in the following we define the fidelity as the normalized process fidelity $F = (F_\chi - 0.25)/0.75$ with a full scale between 0 and 1.

1. The intrinsic error comes from the fact that the lowest-order binomial code implemented in this experiment can only protect quantum information against a single-photon-loss error, therefore the errors that are not in the error set $\{\hat{I}, \hat{a}_c\}$ would cause a failure of the QEC operation. These errors include multiple-photon-loss errors, the photon gain error due to thermal excitations, the dephasing errors due to the combination of photon jumps and the self-Kerr effect, and the non-unitary no-jump evolution. Note that the self-Kerr effect can be eliminated by the PASS method as previously discussed. In order to quantify the contribution of these errors, we perform numerical simulations of the QEC process with including only the cavity decay and Kerr terms but without the auxiliary qubit decoherence. In the simulation, we also add PASS drives to include Kerr cancellation and consider the cavity relaxation errors during the error detection in each QEC cycle. Besides, we also add an auxiliary qubit excitation error of about 1% for each PASS drive to include the PASS-induced excitation errors [23]. For the one-layer QEC experiment, the simulation gives an intrinsic error of about $\epsilon_{i0} = 6.7\%$ and $\epsilon_{i1} = 5.3\%$ for case 0 (detecting no error with a probability $p_0 = 0.781$) and case 1 (detecting one error with a probability $p_1 = 0.219$), respectively. The simulation of the two-layer QEC experiment gives an intrinsic error of about $\epsilon_{i00} = 12.1\%$, $\epsilon_{i01} = 14.8\%$, $\epsilon_{i10} = 9.2\%$, and $\epsilon_{i11} = 20.4\%$ for case 00 (both detecting no error with a probability $p_{00} = 0.630$), case 01 (first detecting no error and second detecting one error with a probability $p_{01} = 0.152$), case 10 (first detecting one error and second detecting no error with a probability $p_{10} = 0.174$), and case 11 (both detecting one error with a probability $p_{11} = 0.044$), respectively.

2. The parity measurement infidelity is quantified by directly measuring the photon number parities of the cavity

TABLE S2: Error budget for the one- and two-layer QEC processes. *These errors are estimated from numerical simulations.

Parameters		Intrinsic error*	Detection error	Recovery error	Thermal error	Average error	Predicted lifetime	Measured lifetime
1-layer QEC	case 0	6.7%	1.1%	2.7%	0.8%	11.5%	757 μs	755±9 μs
	case 1	5.3%	2.5%	3.9%	0.8%			
2-layer QEC	case 00	12.1%	2.2%	2.7%	1.1%	20.1%	824 μs	805±18 μs
	case 01	14.8%	3.6%	3.9%	1.1%			
	case 10	9.2%	3.6%	6.6%	1.1%			
	case 11	20.4%	5.0%	7.8%	1.1%			

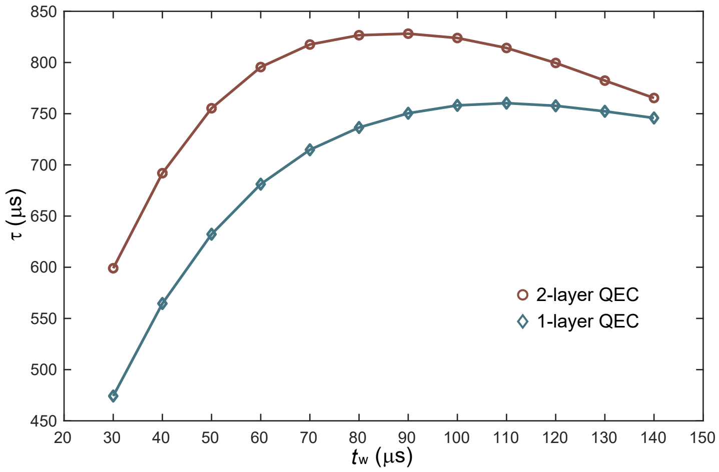

FIG. S8: The numerically estimated lifetimes of both one- and two-layer QECs as a function of the waiting time in each QEC cycle.

states encoded in the six cardinal-point states in the code and error spaces with three consecutive frequency comb parity measurements. The experimental results are shown in Fig. 2(b) in the main text, indicating an average infidelity of $\epsilon_{D_0} = 1.1\%$ and $\epsilon_{D_1} = 2.5\%$ for the cavity states in these two spaces, respectively.

3. The recovery gates for correcting both the single-photon-loss error and no-photon-jump error are implemented by using numerically optimized pulses with the GRAPE method. It is difficult to experimentally calibrate these GRAPE pulses directly because both the initial state preparation and final state measurement also require GRAPE pulses for the encoding and decoding operations. Since the optimization procedure, pulse duration, and hardware are all the same, we assume that the fidelities of all the GRAPE pulses are the same. The gate infidelity of each GRAPE pulse can be roughly estimated from the encoding-decoding process, with a value of $\epsilon_{U_0} = \epsilon_{U_1} = \epsilon_{U_2} = \epsilon_{U_3} = 2.7\%$. After detecting the auxiliary qubit in the excited state, an unconditional π pulse is applied to reset the qubit before the GRAPE operations and results in an error of about 0.7% from the qubit process fidelity with zero idling time. Note that the auxiliary qubit also has a relaxation error of about 0.5% during the feedback latency when detected in the excited state. Thus these two contributions give a total error of about $\epsilon_\pi = 1.2\%$ for the reset π pulse.

4. The auxiliary qubit thermal excitation errors come from the small probability of the excitation of the auxiliary qubit to the $|e\rangle$ state during the total cycle time of about $T_w \approx 92$ μs and $T_w \approx 184$ μs for one- and two-layer QEC, respectively. The estimation from $\epsilon_{\rm th} = n_{\rm th}^{\rm q}(1 - e^{-T_w/T_1^{\rm q}})$ gives average errors of 0.8% and 1.1% for one- and two-layer QEC, respectively.

With all these errors for individual operations in hand, we can calculate the weighted average total errors from the following equation:

$$\begin{aligned} \epsilon_1 &= p_0\left(\epsilon_{i0} + \epsilon_{D_0} + \epsilon_{U_0} + \epsilon_{\rm th}\right) \\ &+ p_1\left(\epsilon_{i1} + \epsilon_{D_1} + \epsilon_{U_1} + \epsilon_\pi + \epsilon_{\rm th}\right), \end{aligned} \tag{S10}$$

for the one-layer QEC experiment, and the following equation:

$$\begin{aligned} \epsilon_2 &= p_{00}\left(\epsilon_{i00} + \epsilon_{D_0} + \epsilon_{D_0} + \epsilon_{U_2} + \epsilon_{\rm th}\right) \\ &+ p_{01}\left(\epsilon_{i01} + \epsilon_{D_0} + \epsilon_{D_1} + \epsilon_\pi + \epsilon_{U_3} + \epsilon_{\rm th}\right) \\ &+ p_{10}\left(\epsilon_{i10} + \epsilon_{D_1} + \epsilon_\pi + \epsilon_{U_1} + \epsilon_{D_0} + \epsilon_{U_2} + \epsilon_{\rm th}\right) \\ &+ p_{11}\left(\epsilon_{i11} + \epsilon_{D_1} + \epsilon_\pi + \epsilon_{U_1} + \epsilon_{D_1} + \epsilon_\pi + \epsilon_{U_3} + \epsilon_{\rm th}\right), \end{aligned} \tag{S11}$$

for the two-layer QEC experiment.

According to a single exponential decay of the QEC process fidelity, we can estimate the decay time τ of the QEC process by

$$\tau = -\frac{T_w}{\ln(1-\epsilon)}. \tag{S12}$$

The predicted lifetimes for both the one- and two-layer QEC experiments are listed in Table S2, as well as the measured lifetimes, which are consistent with each other.

In addition, we also calculate the expected lifetimes of the one- and two-layer QECs as a function of the waiting time of the idle operation, and the results are shown in Fig. S8. In our QEC experiments, we choose an optimal waiting time of about 90 μs to achieve the optimal QEC performance.

---

[1] A. Wallraff and et al., "Strong coupling of a single photon to a superconducting qubit using circuit quantum electrodynamics," Nature **431**, 162 (2004).
[2] A. Blais, R.-S. Huang, A. Wallraff, S. M. Girvin, and R. J. Schoelkopf, "Cavity quantum electrodynamics for superconducting electrical circuits: An architecture for quantum computation," Phys. Rev. A **69**, 062320 (2004).
[3] A. Blais, A. L. Grimsmo, S. M. Girvin, and A. Wallraff, "Circuit quantum electrodynamics," Rev. Mod. Phys. **93**, 025005 (2021).
[4] H. Paik and et al., "Observation of High Coherence in Josephson Junction Qubits Measured in a Three-Dimensional Circuit QED Architecture," Phys. Rev. Lett. **107**, 240501 (2011).
[5] G. Kirchmair and et al., "Observation of quantum state collapse and revival due to the single-photon Kerr effect," Nature **495**, 205 (2013).
[6] B. Vlastakis and et al., "Deterministically Encoding Quantum Information Using 100-Photon Schrödinger Cat States," Science **342**, 607 (2013).
[7] J. Koch and et al., "Charge-insensitive qubit design derived from the Cooper pair box," Phys. Rev. A **76**, 042319 (2007).
[8] M. Reagor and et al., "Quantum memory with millisecond coherence in circuit QED," Phys. Rev. B **94**, 014506 (2016).
[9] C. Wang and et al., "A Schrödinger cat living in two boxes," Science **352**, 1087 (2016).
[10] Y. Y. Gao and et al., "Entanglement of bosonic modes through an engineered exchange interaction," Nature **566**, 509 (2019).
[11] C. Axline and et al., "An architecture for integrating planar and 3D cQED devices," Appl. Phys. Lett. **109**, 042601 (2016).
[12] K. S. Chou and et al., "Deterministic teleportation of a quantum gate between two logical qubits," Nature **561**, 368 (2018).
[13] M. Reagor and et al., "Reaching 10 ms single photon lifetimes for superconducting aluminum cavities," Appl. Phys. Lett. **102**, 192604 (2013).
[14] A. P. M. Place and et al., "New material platform for superconducting transmon qubits with coherence times exceeding 0.3 milliseconds," Nat. Commun. **12**, 1779 (2021).
[15] C. Wang and et al., "Towards practical quantum computers: Transmon qubit with a lifetime approaching 0.5 milliseconds," npj Quantum Inf. **8**, 3 (2022).
[16] F. Motzoi, J. M. Gambetta, P. Rebentrost, and F. K. Wilhelm, "Simple Pulses for Elimination of Leakage in Weakly Nonlinear Qubits," Phys. Rev. Lett. **103**, 110501 (2009).
[17] J. M. Gambetta, F. Motzoi, S. T. Merkel, and F. K. Wilhelm, "Analytic control methods for high-fidelity unitary operations in a weakly nonlinear oscillator," Phys. Rev. A **83**, 012308 (2011).
[18] N. Khaneja, T. Reiss, C. Kehlet, T. Schulte-Herbrüggen, and S. J. Glaser, "Optimal control of coupled spin dynamics: Design of NMR pulse sequences by gradient ascent algorithms," J. Magn. Reson. **172**, 296 (2005).
[19] S. E. Nigg and et al., "Black-Box Superconducting Circuit Quantization," Phys. Rev. Lett. **108**, 240502 (2012).
[20] A. P. Sears and et al., "Photon shot noise dephasing in the strong-dispersive limit of circuit QED," Phys. Rev. B **86**, 180504 (2012).
[21] Q. Guo and et al., "Dephasing-Insensitive Quantum Information Storage and Processing with Superconducting Qubits," Phys. Rev. Lett. **121**, 130501 (2018).
[22] M. H. Michael and et al., "New Class of Quantum Error-Correcting Codes for a Bosonic Mode," Phys. Rev. X **6**, 031006 (2016).
[23] Y. Ma and et al., "Error-transparent operations on a logical qubit protected by quantum error correction," Nat. Phys. **16**, 827 (2020).
[24] L. Hu and et al., "Quantum error correction and universal gate set operation on a binomial bosonic logical qubit," Nat. Phys. **15**, 503 (2019).
